# Epidemiological profile of microbial keratitis in Alexandria-Egypt a 5 years retrospective study

**DOI:** 10.1186/s12348-023-00332-7

**Published:** 2023-04-13

**Authors:** Suzan Ibrahim Sakr, Amira Ahmed Nayel, Christeena Saeed Habeel, Hala Kamal Elkhabiry, Ghada Mahmoud Ibrahim, Mona Mohamed Tolba, Alaa Atef Ghaith

**Affiliations:** 1 Cornea Clinic, Alexandria Ophthalmology Hospital, Ministry of Health and Population of Egypt, Alexandria, Egypt; 2 Clinical Pharmacy Department, Alexandria Ophthalmology Hospital, Ministry of Health and Population of Egypt, Alexandria, Egypt; 3 Microbiology Department, Alexandria Ophthalmology Hospital, Ministry of Health and Population of Egypt, Alexandria, Egypt; 4 Parasitology Department, Medical Research Institute, Alexandria, Egypt; 5 Ophthalmology Department, Faculty of Medicine, Alexandria University, Alexandria, Egypt

**Keywords:** Microbial keratitis/risk factors, Viral keratitis, Microorganisms, Gram-positive bacteria, Filamentous fungi, Contact lens, *Acanthamoeba*

## Abstract

**Objective:**

To evaluate the epidemiologic profile of microbial keratitis in Alexandria- Egypt, with special emphasis on risk factors, visual outcome and microbiological results.

**Methods:**

This retrospective study reviewed files of patients treated for microbial keratitis during a period of 5 years at Alexandria Ophthalmology Hospital Cornea Clinic, Alexandria- Egypt, between February 2017 and June 2022. The patients were evaluated for the risk factors e.g., trauma, eyelid disorders, co-morbidities, and contact lens use. They were also evaluated for their clinical picture, the identified microorganisms, visual outcomes, and complications. Non-microbial keratitis and incomplete files were excluded from the study.

**Results:**

A total of 284 patients were diagnosed as microbial keratitis in our study. Viral keratitis was the most common cause of microbial keratitis (*n* = 118 (41.55%)), followed by bacterial keratitis (*n* = 77 (27.11%)), mixed keratitis (*n* = 51 (17.96%)), acanthamoeba keratitis (*n* = 22 (7.75%)) and the least cause was fungal keratitis (*n* = 16 (5.63%)). Trauma was the most common risk factor for microbial keratitis (29.2%). Fungal keratitis had a statistically significant association with trauma (*p* < 0.001), while the use of contact lenses had a statistically significant association with *Acanthamoeba* keratitis (*p* < 0.001). The percentage of culture-positive results in our study was 76.8%. Gram-positive bacteria were the most frequently isolated bacterial isolate (*n* = 25 (36.2%)), while filamentous fungi were the most frequently isolated fungi (*n* = 13(18.8%)). After treatment, there was a significant increase in the mean visual acuity among all groups; it was significantly higher in *Acanthamoeba* keratitis group with a mean difference of 0.262 ± 0.161 (*p* = 0.003).

**Conclusion:**

Viral keratitis followed by bacterial keratitis were the most frequent etiologic agents causing microbial keratitis found in our study. Although trauma was the most frequent risk factor for microbial keratitis, contact lens wear was found an important preventable risk factor for microbial keratitis in young patients. Performing culture properly whenever indicated before starting antimicrobial treatment increased the cultures’ positive results.

## Introduction

Microbial keratitis (MK) is an infection of the cornea caused by a range of pathogens including bacteria, viruses, parasites (e.g., *Acanthamoeba*), and fungi (yeasts, and filaments). It is considered a potentially sight-threatening disease if improperly managed especially in developing countries [1]. The incidence of this disease varies around the world. In the United States, it is 11 cases per 100.000 inhabitants [2], While in developing countries that number is far bigger, reaching 799 cases per 100.000 inhabitants per year in Nepal [3].

The history of contact lens (CL) wear, ocular trauma, changes in the ocular surface (blepharitis, penetrating keratoplasty, and dry eye), and systemic diseases (diabetes, and rheumatoid arthritis) are the most significant risk factors associated with the onset of MK [4].

The diagnosis of MK is made on the clinical basis together with microbiological evaluation [5]. The microbiological profile of microbial keratitis has shown great differences worldwide. An American study found that in the northern cooler states, bacterial keratitis is more prevalent while in the southern states fungal keratitis is more prevalent [6]. Due to the continuous shifting in microbiological profile and antibiotics resistance profiles reported in several studies, microbiological investigations and antibiotic susceptibility are mandatory to provide an effective treatment [7].

Although MK is one of the main causes of corneal blindness and visual disability, especially in developing countries [8], there is a lack of previous reporting of microbial keratitis epidemiology in our region. This study aimed to characterize the epidemiological profile and the most important risk factors for MK at Alexandria ophthalmology hospital, Alexandria, Egypt.

## Methods

This is a retrospective study of patients diagnosed with microbial keratitis in the period between February 2017 and June 2022 at the cornea clinic in Alexandria Ophthalmology Hospital in Alexandria; a Mediterranean city in Egypt at the western edge of the Nile River. Being a large, specialized hospital in Alexandria, it is considered an important referral centre in Alexandria and the surrounding cities. This study was conducted after approval from the Medical Research Ethics Committee, Ministry of Health and Population of Egypt. The study included patients of both sexes of all ages. Non-microbial keratitis including Mooren's ulcers, chemical burns, and Shield ulcers were excluded. Files with incomplete data and patients lost to follow-up before complete healing were excluded from further analysis.

The relevant data were collected from the hospital’s medical records of patients diagnosed with MK at the cornea clinic, then analysed using the appropriate statistical methods. The collected data included patients’ age, sex, general history of systemic diseases, ocular history of MK {onset, duration of symptoms, history of recurrence}, risk factors (trauma, CL use, and previous history of ocular surgeries). Ophthalmologic examination data included lid examination, visual acuity at the time of presentation and after complete cure, and ulcers features at initial presentation (site, size, and depth). Ulcers’ sites were determined as central (involving the central 4-mm diameter of the cornea) and peripheral ulcers. The ulcer size was classified as small (˂ 2 mm), moderate (2–5 mm), or large (> 5 mm). The density of infiltration, the severity of corneal oedema, hypopyon presence, and keratic precipitates (KPs) presence were documented. Also, Corneal scraping results, the given treatment, and the clinical outcome were recorded. The visual acuity was measured using Snellen’s chart and recorded in decimal notation. Study participants with counting fingers, hand motions, light perception, and no light perception visual acuity were assigned a decimal of 0.02, 0.004, 0.002, and 0, respectively.

### Microbiological investigation protocol

Corneal scraping was ordered according to the American Academy of Ophthalmology recommendations [9]. Under aseptic conditions and after instillation of topical anaesthetic eye drops, corneal scrapes were obtained with a sterile blade 15 aiming at the ulcer edge and floor for in vitro culture. In vitro culture included chocolate agar, blood agar plate (BAP), MacConkey, Sabouraud’s dextrose agar plates (SDA), and brain heart infusion broth enrichment media (BHI). All media were sent to Alex. Ophthalmology Hospital Microbiology Laboratory where they were incubated at 37◦ c for 24 to 48 h. Regarding incubated BHI broth it was inspected for turbidity; turbid broth was sub-cultured on BAP, MacConkey agar plate, and SDA. In case of the presence of growth, the colony morphology was inspected. The disc diffusion method on Mueller Hinton agar was used to conduct an antimicrobial susceptibility test on each identified bacterium. Regarding SDA, it was incubated at 37◦c aerobically and checked for fungal growth every other day for 14 days.

In positive history of contact lens wear, the lens case was sent to the Medical Research Institute parasitology lab. Swabs from the contact lens, lens case, and lens solution [10] were spread on clean glass slides then fixed with methanol and allowed to air-dry for 5 min. The slides were then stained with Giemsa stain and examined for *Acanthamoeba* trophozoites and cysts with an oil immersion lens.

### Management protocols

**Fig. 1:**
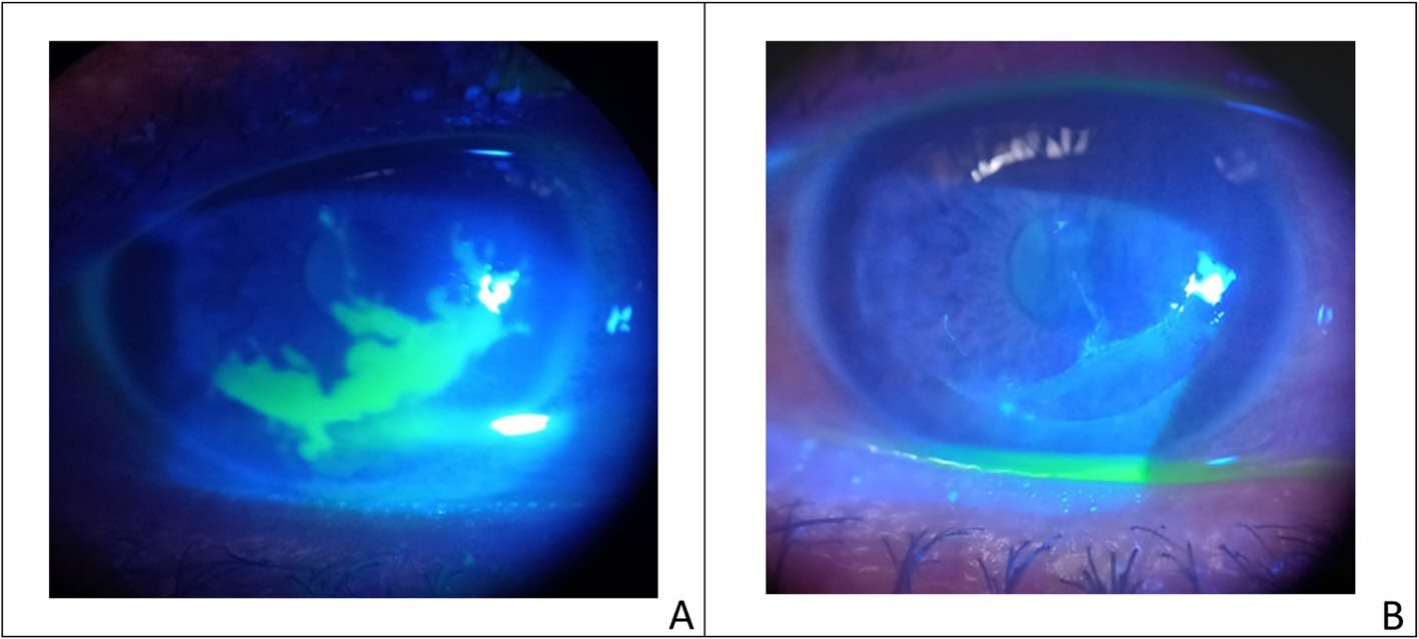
A case of geographic ulcer post- LASIK surgery; pre (**A**) and post treatment (**B**)

Viral keratitis diagnosis and treatment were based on their typical clinical appearance and/or previous ocular history, they did not require any microbiological investigation. Epithelial keratitis cases received topical antiviral for 10 days which increased in geographic ulcers to 14 days (Fig. 1). Stromal keratitis, and endotheliitis cases received systemic antiviral acyclovir 400 mg 5 times daily and topical steroids 5 times daily for one week with gradual tapering till oedema subsided. In stromal keratitis combined with epithelial defect, steroids were not given until complete epithelial healing occurred. In neurotrophic ulcers, systemic doxycycline 100 mg was given twice daily for 1 month together with preservative-free artificial tears every 2 h, while autologous serum was used in resistant cases. In herpes zoster ophthalmicus (HZO), the dose of systemic antiviral increased to 800 mg 5 times daily for 2 weeks.

**Fig. 2:**
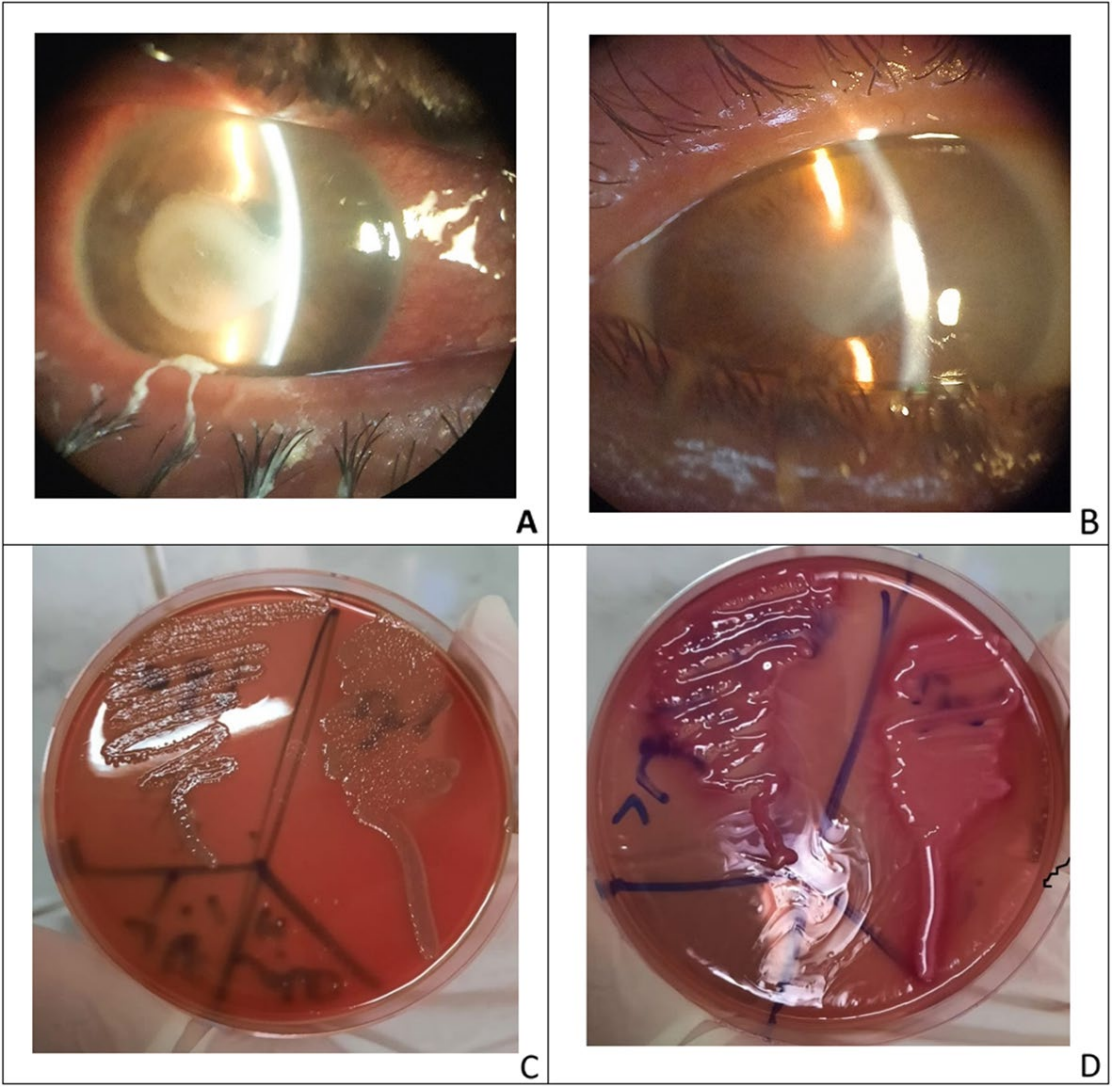
A case of bacterial keratitis Klebsiella spp. pre (**A**), and post-treatment (**B**), non-hemolytic medium size colonies growth on blood agar (**C**), large mucoid pink colonies growth on MacConkey agar (**D**)

Bacterial keratitis cases were divided into non-sight-threatening and sight-threatening keratitis. Non-sight-threatening keratitis with small, superficial, off-axis lesions with infiltrate size of 2 mm or less received empirical treatment according to a standard protocol with moxifloxacin monotherapy, a fourth-generation fluoroquinolone broad-spectrum antibiotic [11]. Sight-threatening bacterial keratitis characterized by medium or large size ulcers, deep infiltration, rapid progression within 3 days, presence of hypopyon or involving visual axis, treatment with topical fortified vancomycin and fortified gentamycin was given to cover both Gram-positive and Gram-negative pathogens (Fig. 2). Drops were given every hour for the first few days to achieve therapeutic tissue concentrations and rapid control of the infection, then the frequency was reduced later based on the clinical response [9]. Topical steroid was contraindicated until complete cure. Oral or parenteral antibiotics were used only in ulcers with perforation, scleral involvement, or endophthalmitis. Treatment was modified primarily by the clinical response taking into consideration the results of cultures and sensitivity testing, especially if the patient is not responding to initial therapy. Cases with blepharitis were treated with topical azithromycin twice daily with lid hygiene and in severe cases, systemic doxycycline was added twice daily.

**Fig. 3:**
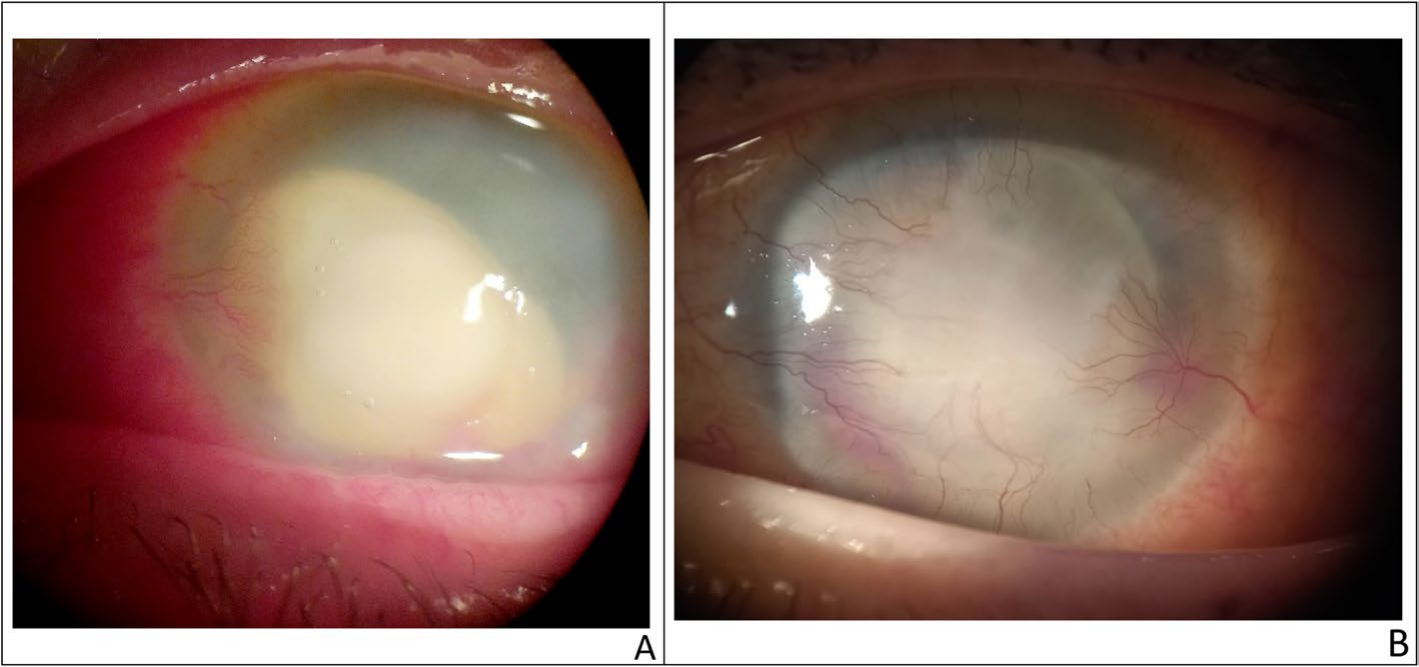
A case of severe fungal keratitis pre (**A**), and post treatment with voriconazole fortified eye drops and systemic itraconazole (**B**)

Fungal keratitis diagnosis was based on the history of trauma or exposure to vegetable matter, the clinical presentation of raised or grey ulcers, satellite or multiple lesions, feathery edges, thick hypopyon, and lab results. The standard approach to treatment in mild cases is topical natamycin 5% every hour in combination with prophylactic fourth-generation fluoroquinolone 5 times daily. Modification of treatment was done in cases not responding to natamycin. Considering microbiological results, amphotericin B 0.15% was added in candida spp and voriconazole was administered in resistant cases. In severe cases with severe stromal infiltrate and thick hypopyon, systemic itraconazole 100 mg was added to topical treatment twice daily for 10 days (Fig. 3). Treatment was continued with a gradual decrease in frequency according to the activity of keratitis till the resolution occurred; one month in mild cases and 3 months in severe cases [12].

**Fig. 4:**
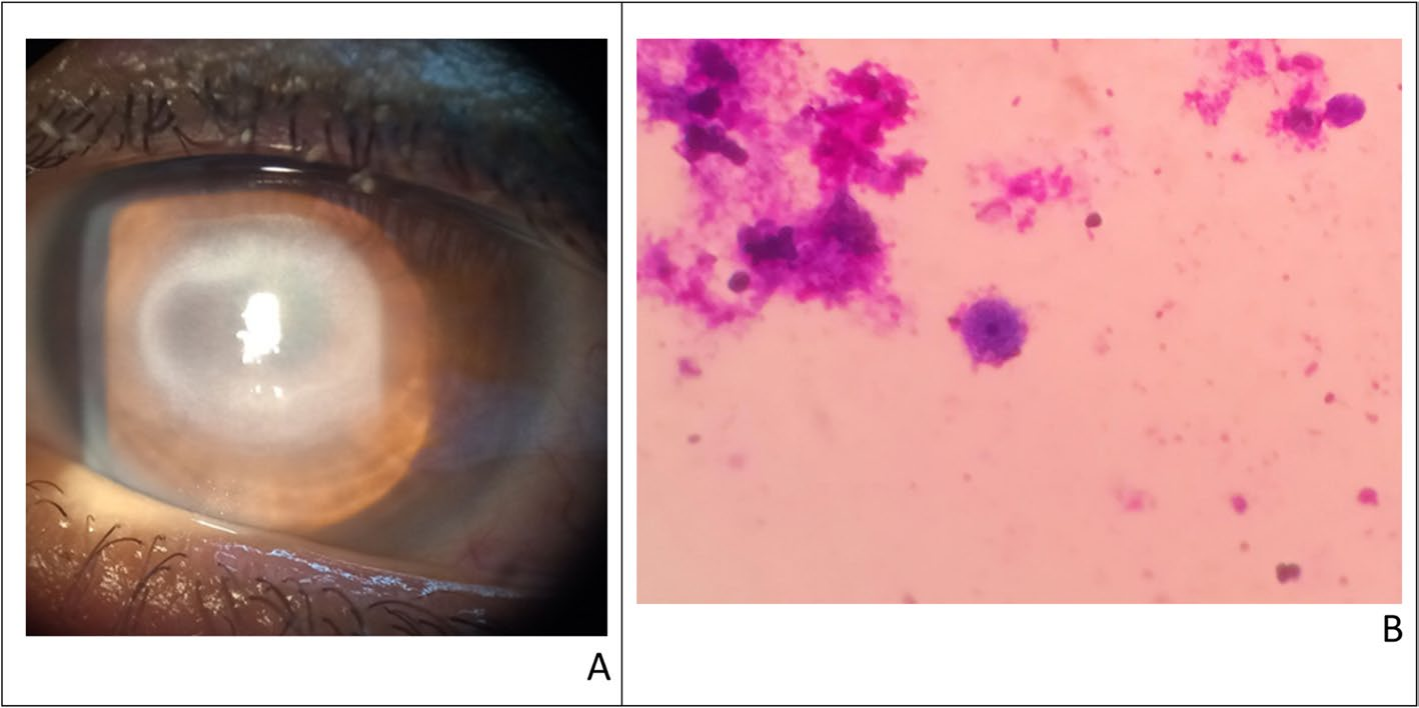
A case of severe acanthamoeba keratitis presented with immune ring (**A**), Acanthamoeba cyst stained by Giemsa stain (**B**)

*Acanthamoeba* keratitis cases were divided into mild and severe keratitis. Mild cases with epitheliopathy and radial keratoneuritis were treated using polyhexamethylene biguanide drops every hour around the clock for the first few days of treatment with gradual tapering of drops depending on clinical response, while in severe cases with ring infiltration combined therapy with polyhexamethylene biguanide and propamidine 0.1% was given [13] (Fig. 4). Medications were continued for 3 months in mild cases and 6 months in severe cases to prevent relapses.

**Fig. 5:**
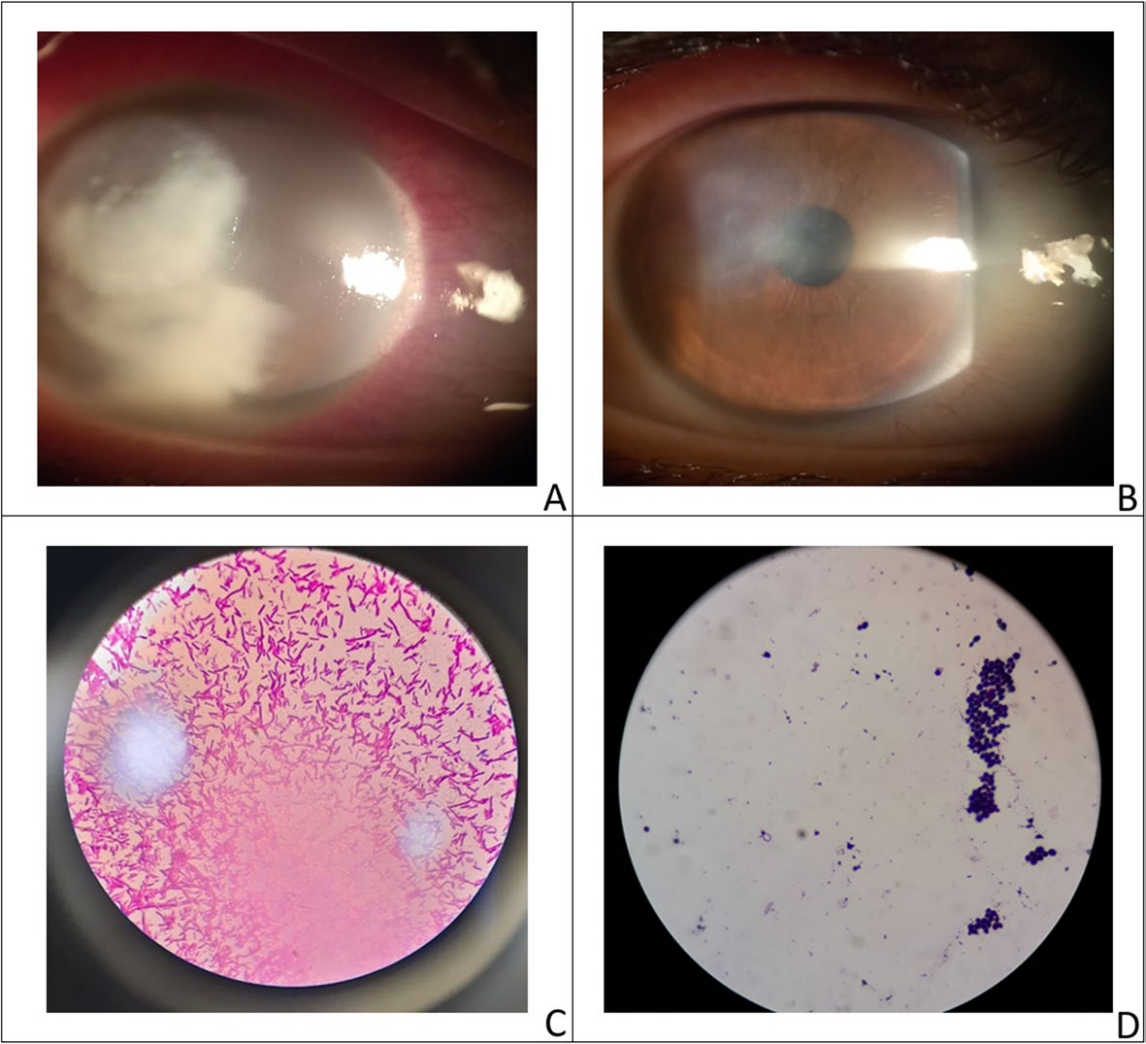
A case of mixed bacterial and fungal keratitis pre (**A**), and post treatment (**B**), Gram negative bacilli; Pseudomonas (magnificationX1000) (**C**), budding yeast cells; Candida spps (magnificationX1000) (**D**)

Mixed keratitis was diagnosed if two or more types of microorganisms were simultaneously present during the same infective episode (Fig. 5). Treatment was adjusted according to the clinical picture and lab results.

General lines of therapy in impending perforation or perforated cases included systemic doxycycline 100 mg twice daily, systemic vitamin C 1 g twice daily, antiglaucoma eye drops of beta blockers or carbonic anhydrase inhibitors (CAI), and cycloplegics.

Data were fed to the computer and analyzed using IBM SPSS software package version 20.0. (Armonk, NY: IBM Corp). They were tested for normality by the Shapiro–Wilk test Categorical data were represented as numbers and percentages. Chi-square test was applied to compare two groups. Alternatively, Monte Carlo and Fisher Exact correction test was applied when more than 20% of the cells have an expected count of less than 5, while ANOVA was used for comparing the studied groups and followed by Post Hoc test (Tukey) for pairwise comparison. Kruskal Wallis test was used to compare different groups for non-normally distributed quantitative variables and followed by Post Hoc test (Dunn's for multiple comparisons test) for pairwise comparison, Wilcoxon signed ranks test for non-normally distributed quantitative variables, to compare between two periods. The significance of the obtained results was judged at the 5% level.

Superscript letters in the illustrating tables were added to the values of the different studied groups. Values with different superscript letters have a statistically significant difference, while those with similar superscript letters does not have a statistically significant difference.

## Results

A total of 585 patients were diagnosed as keratitis during the study period. Three hundred and one patients were excluded and 284 patients with microbial keratitis were included. The collected data were divided according to the causative organisms into 5 groups: viral, bacterial, fungal, acanthamoeba, and mixed keratitis.

**Fig. 6:**
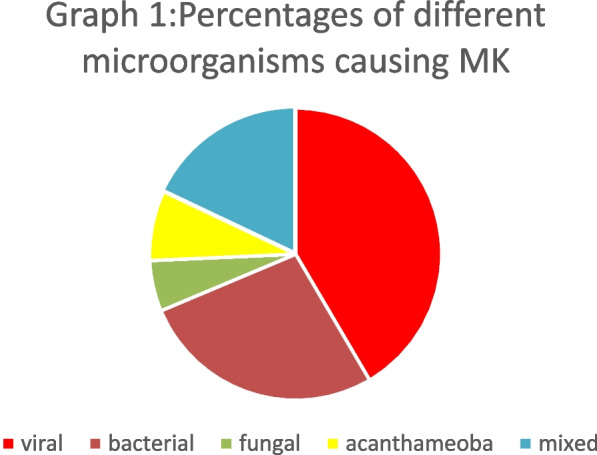
The percentage of distribution of the different microorganisms causing MK

Viral keratitis was the most common cause of microbial keratitis (118 cases- 41.55%) followed by bacterial keratitis (77 cases -27.11%), mixed keratitis (51 cases -17.96%), and acanthamoeba (22 cases -7.75%). The least cause was fungal keratitis (16 cases -5.63%) (Fig. 6).

### Demographic data

The age of the studied population ranged from 2.5 to 88 years old. The mean age was 40.4 years. The mean age of the *Acanthamoeba* group was significantly younger than the other groups (23.2 years, *p* < 0.001) (Table 1).

**Table 1:** Comparison between MK groups according to age

	**Viral (*n* = 118)**	**Bacterial (*n* = 77)**	**Fungal (*n* = 16)**	**Acanthamoeba (*n* = 22)**	**Mixed (*n* = 51)**	**Total (*n* = 284)**	***p***
**Age (years)**							
Mean ± SD	46.8^a^ ± 18.5	41^a^ ± 23	49^a^ ± 18.5	23.2^b^ ± 6.9	44.2^a^ ± 18	40.4 ± 17	< 0.001^*^
Median (Min. – Max.)	49.5(4 – 88)	38(2.5 – 85)	50(10 – 74)	21 (14 – 37)	45(4 – 78)	45(2.5 –88)	

*p*: *p*-value for comparing between the studied groups, *SD* Standard deviation

^*^Statistically significant at *p* ≤ 0.05 Numbers with different letters are significant

**Fig. 7:**
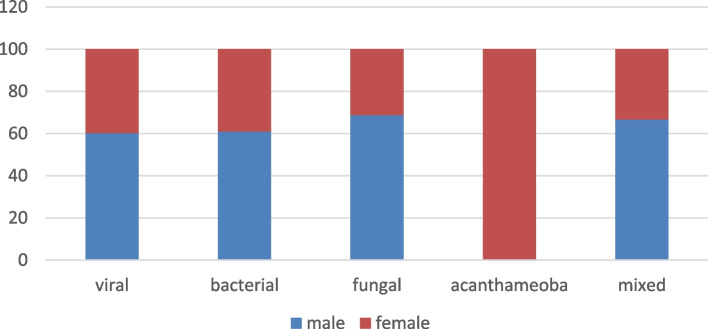
The distribution of cases according to sex

Of the 284 patients, 163 cases (57.4%) were males and 121 cases (42.6%) were females. In the acanthamoeba group, all cases were female and this was statistically significant in comparison with the other groups (*p* < 0.001) (Fig. 7)

Of the 284 patients, 163 cases (57.4%) were males and 121 cases (42.6%) were females. In the acanthamoeba group, all cases were female and this was statistically significant in comparison with the other groups (*p* < 0.001) (Fig. 7).

### Risk factors

Ocular trauma was the most common predisposing factor for microbial keratitis. It occurred in 83 cases (29.2%). Thirty-three cases (11.6%) had a history of contact lens wearing. *Acanthamoeba* keratitis had a statistically significant association with contact lens wearing (100%) (p < 0.001). Of the 284 studied cases, 35 had blepharitis (12.3%), which was significantly higher in the bacterial group (24 cases-31.2% of all bacterial keratitis patients) (p < 0.001). Ocular surgery and diabetes mellitus were found non-significant risk factors (Table 2).

**Table 2:** Comparison between MK groups according to risk factors

	**Viral (*n* = 118)**	**Bacterial (*n* = 77)**	**Fungal (*n* = 16)**	**Acanthamoeba (*n* = 22)**	**Mixed (*n* = 51)**	**Total (*n* = 284)**	^MC^*p*
Trauma	9^a^ (7.6%)	40^b^ (51.9%)	16^c^ (100%)	4^ad^ (18.2%)	14^d^ (27.5%)	83(29.2%)	< 0.001^*^
Contact lens	0^a^ (0%)	6^b^ (7.8%)	0^ab^ (0%)	22^c^ (100%)	5^b^ (9.8%)	33(11.6%)	< 0.001^*^
Blepharitis	7^a^ (5.9%)	24^b^ (31.2%)	0^a^ (0%)	0^a^ (0%)	4^a^ (7.8%)	35(12.3%)	< 0.001^*^
Ocular surgery	5 (4.2%)	7 (9.1%)	3 (18.8%)	0 (0%)	3 (5.9%)	18(6.3%)	0.128
DM	19 (16.1%)	11 (14.3%)	4 (25%)	2 (9.1%)	8 (15.7%)	44(15.5%)	0.757

*MC* Monte carlo, *p*: *p*-value for comparing between the studied groups

^*^Statistically significant at *p* ≤ 0.05 Numbers with different letters are significant

### Clinical results

- **Onset duration and Cure duration**

The time between the onset of complaints and examination was different among groups. We found that most cases with bacterial keratitis (43 -55.8%) came within the first week of complaints and this was statistically significant (*p* < 0.001). It was also founded that 80 cases (67.8%) among the herpetic group and 28 cases (54.9%) of the mixed group came for ocular examination between one week to one month. Most *Acanthamoeba* keratitis cases (10 -45.5%) and fungal keratitis cases (7 -43.8%) had a statistically significant delayed referral (more than one month) (*p* < 0.001).

After the exclusion of 69 cases that failed to show up from further analysis, we found that the cure duration in most cases with bacterial keratitis (36 -62.1%)) and viral keratitis (56 -56.6%)) was 2 weeks or less and this was statistically significant (*p* = 0.011). The cure duration was longer in the fungal group (7 -87.5%) and in the mixed group (25 -65.8%) where it reached more than 2 weeks (Table 3).

- **Visual acuity before and after treatment**

**Table 3:** Comparison between MK groups according to onset duration and Cure duration

	**Viral**	**Bacterial**	**Fungal**	**Acanthamoeba**	**Mixed**	***p***
**Onset duration**	**(*n* = 118)**	**(*n* = 77)**	**(*n* = 16)**	**(*n* = 22)**	**(*n* = 51)**	
< 1w	24^a^ (20.3%)	43^b^ (55.8%)	0^c^ (0%)	6^a^ (27.3%)	17^a^ (33.3%)	< 0.001^*^
1w-1 m	80^a^ (67.8%)	28^b^ (36.4%)	9^ab^ (56.3%)	6^b^ (27.3%)	28^a^ (54.9%)	
> 1 m	14^a^ (11.9%)	6^a^ (7.8%)	7^b^ (43.8%)	10^b^ (45.5%)	6^a^ (11.8%)	
**Cure duration**	**(*n* = 99)**	**(*n* = 58)**	**(*n* = 8)**	**(*n* = 12)**	**(*n* = 38)**	
< 2w	56^a^ (56.6%)	36^a^ (62.1%)	1^b^ (12.5%)	6^ab^ (50%)	13^b^ (34.2%)	^MC^*p* = 0.011^*^
2w-1 m	33^ab^ (33.3%)	14^b^ (24.1%)	7^c^ (87.5%)	6^abc^ (50%)	18^a^ (47.4%)	
> 1 m	10^a^ (10.1%)	8^a^ (13.8%)	0^a^ (0%)	0^a^ (0%)	7^a^ (18.4%)	

Numbers with different letters are significant

*MC* Monte carlo, *p*: *p*-value for comparing between the studied groups

^*^Statistically significant at *p* ≤ 0.05

There was a significant increase in the mean visual acuity among all groups. The *Acanthamoeba* group showed the largest gain in visual acuity (mean difference of 0.262 ± 0.161) while the mixed group showed the least gain (mean difference of 0.098 ± 0.155). The *Acanthamoeba* group was associated with the best mean final visual acuity (0.400 ± 0.191) (Table 4). We excluded 11 paediatric patients because their vision couldn’t be documented and 69 cases that failed to follow up, hence the difference in the number of eyes before and after treatment.

- **Corneal features**

**Table 4:** Comparison between the MK groups according to visual acuity

Visual acuity (decimal)	Viral	Bacterial	Fungal	Acanthamoeba	Mixed	*p*
**Before**	**(*n* = 116)**	**(*n* = 69)**	**(*n* = 16)**	**(*n* = 22)**	**(*n* = 50)**	0.004^*^
Mean ± SD	0.078 ± 0.137	0.085 ± 0.127	0.017 ± 0.018	0.100 ± 0.133	0.037 ± 0.058	
Median (Min. – Max.)	0.020^a^ (0.0 – 0.700)	0.020^ab^ (0.0 – 0.500)	0.010^c^ (0.0 – 0.050)	0.020^a^ (0.010 – 0.400)	0.010^bc^ (0.0 – 0.300)	
**After**	**(*n* = 97)**	**(*n* = 50)**	**(*n* = 8)**	**(*n* = 12)**	**(*n* = 37)**	0.003^*^
Mean ± SD	0.234 ± 0.070	0.229 ± 0.256	0.176 ± 0.216	0.400 ± 0.191	0.132 ± 0.172	
Median (Min. – Max.)	0.100^b^ (0.0 – 1.000)	0.130^bc^ (0.0 – 0.800)	0.040^bc^ (0.0 – 0.500)	0.400^a^ (0.100 – 0.600)	0.100^b^ (0.0 – 0.700)	
** Mean difference**	0.157 ± 0.176	0.136 ± 0.161	0.158 ± 0.202)	0.262 ± 0.161	0.098 ± 0.155	
** p**_**1**_	< 0.001^*^	< 0.001^*^	0.018^*^	0.002^*^	< 0.001^*^	

Numbers with different letters are significant

*MC* Monte carlo, *p*: *p*-value for comparing between the studied groups

^*^Statistically significant at *p* ≤ 0.05 p1: *p*-value for Wilcoxon signed ranks test for comparing between before and after in each group

The absence of ulcer was found to be significantly associated with viral and *Acanthamoeba* groups (*p* < 0.001). Central ulceration was present in 125 cases (44%). Medium-sized ulcers showed a statistically significant association with fungal and mixed MK groups. (*p* < 0.001) Superficial ulceration was present in 179 cases (63%), while there was a statistically significant absence of corneal perforation in the fungal and the *Acanthamoeba* groups (Table 5).

**Table 5:** Comparison between MK groups according to baseline characteristics of corneal ulcers’ site, size, and depth

	**Viral (*n* = 118)**	**Bacterial (*n* = 77)**	**Fungal (*n* = 16)**	**Acanthamoeba** **(*n* = 22)**	**Mixed (*n* = 51)**	**Total (*n* = 284)**	***p***
**No ulcer**	69^a^ (58.5%)	0^b^ (0%)	0^b^ (0%)	12^a^ (54.5%)	0^b^ (0%)	81(28.5%)	
**Ulcer Site**							
Central	34^a^ (28.8%)	42^b^ (54.5%)	13^c^ (81.2%)	4^a^ (18.2%)	32^bc^ (62.7%)	125 (44%)	< 0.001^*^
Peripheral	15^a^ (12.7%)	35^b^ (45.5%)	3^ac^ (18.8%)	6^abc^ (27.3%)	19^bc^ (37.3%)	78(27.5%)	
**Ulcer Size (mm)**							
Small	19^a^ (16.1%)	47^b^ (61%)	5^ac^ (31.2%)	6^ac^ (27.3%)	24^bc^ (47.1%)	101 (35.6%)	^MC^*p* < 0.001^*^
Medium	23^a^ (19.5%)	19^a^ (24.7%)	10^b^ (62.5%)	4^a^ (18.2%)	22^b^ (43.1%)	78 (27.5%)	
Large	7^a^ (5.9%)	11^b^ (14.3%)	1^ab^ (6.2%)	0^ab^ (0%)	5^ab^ (9.8%)	24 (8.5%)	
**Ulcer Depth**							
One third	42^a^ (35.6%)	68^b^ (88.3%)	14^b^ (87.5%)	10^a^ (45.5%)	45^b^ (88.2%)	179(63%)	^MC^*p* < 0.001^*^
Two thirds	5^a^ (4.2%)	5^a^ (6.5%)	2^a^ (12.5%)	0^a^ (0%)	4^a^ (7.8%)	16(5.6%)	
Perforated	2^a^ (1.7%)	4^a^ (5.2%)	0^a^ (0%)	0^a^ (0%)	2^a^ (3.9%)	8(2.8%)	

Numbers with different letters are significant

*MC* Monte carlo, *p*: *p*-value for comparing between the studied groups

^*^Statistically significant at *p* ≤ 0.05

Regarding the infiltration, it was found that the viral group was significantly associated with absence and minimal infiltration. On the other hand, bacterial, fungal, and mixed MK groups were significantly associated with dense infiltration (*p* < 0.001). KPs were significantly associated with viral and mixed groups 72 cases (25.4%) (*p* < 0.001). Hypopyon was present in 45 cases (15.8%), and it was significantly absent in the viral and in the *Acanthamoeba* groups (*p* < 0.001) (Table 6).

**Table 6:** Comparison between MK groups according to baseline characteristics of specific corneal features

	**Viral (*n* = 118)**	**Bacterial (*n* = 77)**	**Fungal (*n* = 16)**	**Acanthamoeba** **(*n* = 22)**	**Mixed (*n* = 51)**	**Total (*n* = 284)**	***p***
**Infiltration**							
Negative	72^a^ (61%)	0^b^ (0%)	0^b^ (0%)	0^a^ (0%)	0^b^ (0%)	72(25.4%)	^MC^*p* < 0.001^*^
1 +	46^a^ (39%)	44^b^ (57.1%)	3^a^ (18.8%)	8^ab^ (36.4%)	19^a^ (37.3%)	120(42.3%)	
2 +	0^a^ (0%)	22^b^ (28.6%)	9^c^ (56.2%)	8^bc^ (36.4%)	28^c^ (54.9%)	67(23.6%)	
3 +	0^a^ (0%)	11^bc^ (14.3%)	4^bc^ (25%)	6^c^ (27.3%)	4^b^ (7.8%)	25(8.8%)	
**Edema**							
Negative	17^a^ (14.4%)	13^a^ (16.9%)	0^a^ (0%)	4^a^ (18.2%)	3^a^ (5.9%)	37(13%)	^MC^*p* = 0.031^*^
Mild	34^a^ (28.8%)	30^a^ (39%)	6^a^ (37.5%)	4^a^ (18.2%)	12^a^ (23.5%)	86(30.1%)	
Moderate	47^a^ (39.8%)	27^a^ (35.1%)	8^ab^ (50%)	14^b^ (63.6%)	24^ab^ (47.1%)	120(42.3%)	
Severe	20^ab^ (16.9%)	7^bc^ (9.1%)	2^abc^ (12.5%)	0^c^ (0%)	12^a^ (23.5%)	41(14.4%)	
**KPs**	61^a^ (51.7%)	0^b^ (0%)	0^b^ (0%)	0^b^ (0%)	11^c^ (21.6%)	72(25.4%)	< 0.001^*^
**Hypopyon**	0^a^ (0%)	19^b^ (24.7%)	8^c^ (50%)	0^a^ (0%)	18^bc^ (35.3%)	45(15.8%)	< 0.001^*^

Numbers with different letters are significant

*MC* Monte carlo, *p*: *p*-value for comparing between the studied groups

^*^Statistically significant at *p* ≤ 0.05

### Viral keratitis

One hundred and fifteen cases (97.5%) were caused by herpes simplex virus while only 3 cases (2.5%) were caused by herpes zoster virus. Stromal keratitis was the most common presentation of HSV (71 cases -60.2%). Bilateral herpes simplex keratitis occurred in only 3 cases (2.5%) (Table 7).

**Table 7:** The distribution of different clinical presentations of viral keratitis

Viral keratitis (*n* = 118)	Clinical presentations	n (%)	Bilaterality
HSV **(*n* = 115)** 97.5%	Dendritic ulcer	22 (18.7%)	3 (2.5%)
	Geographic ulcer	4 (3.4%)	
	Stromal keratitis	71 (60.2%)	
	Ulcer + stromal keratitis	11 (9.3%)	
	Neurotrophic ulcer	7 (5.9%)	
HZV **(*n* = 3)** 2.5%	Nummular keratitis	2 (1.7%)	0
	Micro dendrites	1 (0.8%)	

### Microbiological profile

Corneal scraping for culture and sensitivity was indicated in 69 cases out of a total of 144 cases of bacterial, fungal, and mixed keratitis. Fifty-three cultures were positive (76.8%), whereas only 16 cultures (23.2%) revealed no growth. The laboratory culture demonstrated the growth of bacteria in 40 cases (58%). Among the Gram-positive bacteria, the most frequent organisms were the *Coagulase-negative Staphylococci* (CoNS) (10 cultures (14.5%)). Among the Gram-negative bacteria, *P.aeruginosa* was the most frequent etiologic agent (9 cultures (13%)). Regarding fungi, it was present in 13 cultures (18.8%). Filamentous fungi were the most frequent fungal isolate (8 cultures (11.6%)). Six patients (8.7%) had combined infections of mixed fungi and bacteria (Table 8). Thirty-four cases receiving antimicrobial therapy and 41 non-indicated cases were excluded from doing corneal scraping and culture.

**Table 8:** The distribution of microbial isolates

**Microbial Isolate**	**(*n* = 69)**			**n (%)**
**Bacteria**	40 (58%)	Gram-positive	CoNS	10 (14.5%
			*S. aureus*	8 (11.6%)
			*S. epidermidis*	1 (1.4%)
			*S. pneumonia*	6 (8.7%)
		Gram-negative	*P.aeruginosa*	9 (13%)
			*E. coli*	4 (5.8%)
			*Klebsiella spp.*	2 (2.9%)
**Fungi**	13 (18.8%)	Filamentous		8 (11.6%)
		Candida		5 (7.2%)
**Sterile**	16 (23.2%)			16 (23.2%)

*CoNS* Coagulase-negative Staphylococci, *S. aureus* Staphylococcus aureus, *S. epidermidis* Staphylococcus epidermidis, *E. coli* Escherichia Coli

In suspected cases of *Acanthamoeba* with positive history of CL wear, cytological detection of *Acanthamoeba* trophozoites and cysts from CL, lens cases, and lens-cleaning solutions was done. Among the 22 *Acanthamoeba* cases, fifteen CL cases were investigated for *Acanthamoeba*; eight of them were positive (53.3%).

### Fate and complications

**Fig. 8:**
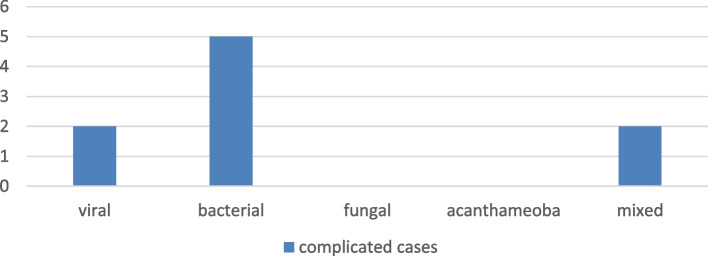
The distribution of complicated cases among the 5 MK groups

Complications were encountered in 9 cases (4.2%). Five complications (2.3%) were in the bacterial group, whereas the viral and the mixed group each had 2 complicated cases (0.9%). Five patients had progressive corneal thinning and corneal perforation, one case ended by endophthalmitis, and two cases ended by corneal melting. Two cases were referred for penetrating keratoplasty and one case required tarsorrhaphy (Fig. 8).

## Discussion

Microbial keratitis (MK) is considered a major cause of visual loss worldwide. Understanding its epidemiology, risk factors, etiological agents, and clinical characteristics will help to reach an accurate diagnosis and in turn proper management. MK varies demographically, and hence, regular regional updates become important. Our study was conducted aiming to describe the latest update of the epidemiological profile of MK in Alexandria-Egypt.

In our study, viral keratitis was the most common cause of microbial keratitis (*n* = 118–41.55%). Similarly, the Asia Cornea Society Infectious Keratitis (ACSIKS) study demonstrated that viral keratitis represented the most common cause (*n* = 434–46%) of MK in China (HSK 24% and HZO 17%) [14]. In our study, 115 cases (97.5%) were caused by herpes simplex virus and only 3 cases (2.5%) were caused by herpes zoster virus. The higher incidence of HZO in China as published by the ACSIKS study may be a reflection of the referral pattern to the ophthalmology centers included in this study. Another two studies, conducted in Menoufia -Egypt and China observed that 15% and 21% of MK, respectively, were caused by herpetic keratitis [15, 16]. The reason for the variation may be due to the climate differences between Alexandria and Menoufia; Alexandria has a cooler climate compared to the warmer climate in Menoufia as it is located in the South Nile Delta of Egypt. The reported incidence of bilateral herpetic keratitis in the literature varies from 1.3% to 12% depending on the diagnosing criteria [17]. In our study, the incidence of bilateral cases was low as it occurred in only 3 cases (2.5%) of HSV.

An important issue associated with Herpetic keratitis is neurotrophic keratopathy (NK). NK can result in poor corneal healing, increased risk of further MK, and other corneal complications such as melting and perforation [18]. NK occurred in 7 cases of total herpetic keratitis and was responsible for the only 2 complicated cases in the viral group.

MK affects individuals across all age groups, especially people aged between 30 and 55 years [19–22]. This is attributed to the underlying risk factors such as ocular trauma associated with the working age group. In our study, we observed that the mean age was in the fifth decade in all groups except for *Acanthamoeba* where the mean age was in the third decade. Similarly, the studies of Tong et al. and Stapleton et al. reported that patients affected by CL-related MK were usually between 25 and 40 years old [23, 24].

Interestingly, many studies have reported that CL-related MK has been shown to exhibit a female predominance of 57–69% [25], and that was similar to our results, as all CL wearers (100%) were female. Except for the Acanthamoeba group, there is a high male prevalence in all MK groups like other studies of MK in South America [26], Asia [14], and Africa [27] reported male prevalence, ranging from 58 to 75%.

Ocular trauma was the most common predisposing factor for microbial keratitis in our study; it occurred in 83 cases of the total cases (29.2%). Likewise, Srinivasan et al. [1] and Keay et al. [28] also found that the most predisposing factor for microbial keratitis was corneal trauma in 65.4%, and 36.4%, respectively. Blepharitis was significantly higher in the bacterial group (*n* = 24–31.2%)). Schaefer et al. [29] also reported blepharitis as a predisposing factor for bacterial keratitis in 21% of cases. Other risk factors e.g., ocular surgery and diabetes, showed non-significant relationship. Similar findings were reported by Keay et al. [28].

In our study, thirty-three cases (11.6%) were contact lens wearers, denoting that CL wear is becoming an important risk factor, mainly due to increasing urbanization as was the case in Taiwan [30]. *Acanthamoeba* keratitis (AK) is highly related to CL wearing and poor lens hygiene especially if washing of lenses with tap water occurred. Al-Herrawy et al. isolated Acanthamoeba spp. from finished water samples in Egypt [31] and it is not surprising that Acanthamoeba organisms have been cultured from lens cases and saline cleaning solutions [32]. Early detection and diagnosis with AK characteristic clinical picture are critical to the outcome of its clinical course [33, 34]. Ulceration in AK does not occur until very late in the disease process. Also, 29 to 49% only of AK cultured cases have a positive result [35, 36]. Hence, in our study, we depended on the cytological detection of acanthamoeba trophozoites and cysts from CL cases. It has the advantage of being fast, easily performed, and readily available in most facilities [37]. Although a positive detection of acanthamoeba in the lens case does not confirm the diagnosis, it highly suggests it [38].

In bacterial keratitis, 43 cases (55.8%) presented within the first week of complaints. Our finding is similar to the findings of Omar et al. and Wong et al. who reported a mean presenting time of 4.67 days [39] and 8.9 days [40], respectively. On the contrary, Toth et al. reported a longer presentation time of 21.3 days [41]. The differences could be due to cultural issues, financial status, awareness, or access to eye care facilities. It was found that about 80 patients (67.8%) among the herpetic group and 28 patients from the mixed group (54.9%) came for ocular examination between one week to one month. Interestingly, most of the delayed referrals (more than one month) was in the Acanthamoeba keratitis group (10 cases (45.5%)), followed by the fungal keratitis group (7 cases (43.8%)). Similarly, the long duration of admission was also reported by Otri et al. [42].

Few studies have prospectively followed patients with microbial keratitis to monitor changes in visual acuity. There was a statistically significant increase in the mean visual acuity among all treated groups in our study. Srinivasan et al. showed that patients with treated bacterial keratitis experience an approximate 2-line improvement in visual acuity from enrolment to 3 weeks [43]. In a prospective study of 273 individuals with presumed microbial keratitis in Nepal, 52.7% experienced ≥ 2 lines of improvement in pinhole visual acuity [44]. Additionally, a study of 30 patients with culture-proven bacterial keratitis found an average visual acuity improvement of 2.5 lines by 10 weeks [45].

A higher proportion of central keratitis was found in this study (61.6%), which is significantly higher in fungal, mixed, and bacterial groups (*p* < 0.001). Similarly, a study in Malysia reported central ulceration in 69% of cases [46]. We found that moderate-to-large ulcers are more likely to occur in fungal keratitis and this was also shown by other investigators [47, 48]. The presence of hypopyon was significantly related to fungal, bacterial, and mixed groups (*p* < 0.001). This agrees with the finding of the study published by Chidambaram et al. They reported aspergillus species and bacterial keratitis were more associated with hypopyon [49].

The percentage of culture-positive results in our study was 76.8%, which was higher than the studies by Otri et al. in the United Kingdom (41%) [42], Omar et al. in Malaysian urban areas (47.5%) [39], and Tananuvat et al. in Thailand (25.6%) [50], and similar to the high rates of culture positivity in studies in the United States (82%) [51] and New Zealand (71%) [40]. Corneal scraping technique, methods of culturing, types of the causative organisms, different types of culturing media, and antibiotic treatment prior to corneal scraping could be the reasons contributing to this variation [39]. The high positivity in our study is attributed to the use of enrichment media (brain heart infusion broth) [52] and the proper scraping technique by well-trained ophthalmologists. An important issue to be mentioned is that the use of antimicrobial eye drops prior to culture was usually associated with negative results. Therefore, culture should be done whenever indicated prior to starting antimicrobial treatment.

Similar to other studies, most of the bacterial keratitis cases were due to Gram-positive organisms [53–55]. Toth et al. and Puig et al. stated that *Coagulase-negative Staphylococci (CoNS)* were the most frequently isolated bacteria [41]. In contrast to our results, another Malaysian study [56] found *Pseudomonas aeruginosa* to be the main causative organism along with other Gram-negative bacteria. In our study, *Pseudomonas aeruginosa* was the most common gram-negative bacteria (13%) similar to a paper published by Toth et al. [41] where *Pseudomonas spp.* was the etiological agent in 10% of cultured cases. This percentage is less than that reported by Norina et al. (40%) [46].

The higher prevalence of bacterial keratitis (27.11%) over that of fungal keratitis (5.63%) in our study contradicted with the Japanese, where a higher prevalence of fungi (50.7%) mainly *Fusarium* was reported [57]. An American study showed that the aetiology depends on the geographic location of the study population, bacterial keratitis was more prevalent in the northern cooler states, while in the southern warmer states and rural areas, fungal infections predominated [6]. This finding corresponds with our results, since our city, Alexandria, is a coastal city.

Since contact lens wearing was found to be a serious preventable risk factor for microbial keratitis, Public Health services should be directed to raising the public awareness of this problem. The role of fever in predisposing attacks of recurrent herpetic keratitis should be furtherly studied among other factors.

The limitations of this study include that it was performed retrospectively. A large number of incomplete medical records were excluded from the study and this was detrimental in limiting the study's sample size. A larger prospective multi-centre study would gather more data to increase the sample size, and this will eventually strengthen our knowledge about the epidemiological profile of microbial keratitis in our region.

## Conclusions

Viral keratitis followed by bacterial keratitis were the most frequent etiologic agents for microbial keratitis found in our study. Although trauma was the most common risk factor for MK, contact lens wearing was found an important risk factor for keratitis in young patients. Proper management of MK increased the post-treatment mean visual acuity among all treated groups. Cytological detection of *Acanthamoeba* trophozoites and cysts in lens cases is an alternative to direct culture from corneal scrape and biopsy. The use of enrichment media (brain heart infusion broth), proper scraping technique, and doing culture whenever indicated prior to starting antimicrobial treatment increased the culture-positive results in our study.

## Data Availability

The datasets used and analyzed during the current study are available from the corresponding author upon reasonable request.

## Abbreviations

MK: Microbial keratitis
BAP: Blood agar plate
SDA: Sabouraud’s dextrose agar plates
BHI: Brain heart infusion broth
MSA: Mannitol salt agar
KPs: Keratic precipitate
CoNS: *Coagulase* *-negative Staph* *ylococci*
HSV: Herpes simplex virus
HZO: Herpes zoster ophthalmicus
CL: Contact lens
